# Amblyopia in a Young Child With an Atypical Response to Treatment: A Case Report

**DOI:** 10.7759/cureus.79257

**Published:** 2025-02-18

**Authors:** Alejandro M Perez, Carlos E Mendoza-Santiesteban, Ta Chen Peter Chang

**Affiliations:** 1 Ophthalmology, Bascom Palmer Eye Institute, University of Miami Health System, Miami, USA

**Keywords:** case report, foveal hypoplasia, genetic testing, isometropic amblyopia, ocular albinism

## Abstract

Amblyopia is a prevalent and treatable cause of visual impairment in children, often responding well to timely intervention. Clinicians should investigate potential subtle structural abnormalities when expected improvements are not achieved. We present the case of a three-year-old blonde male with decreased vision and high astigmatism, identified during routine screening. Initial visual gains with spectacle correction suggested isometropic amblyopia; however, limited improvement over time led to further investigation. Optical coherence tomography (OCT) revealed abnormal foveal contour, and light fundus pigmentation raised suspicion of ocular albinism (OA), a diagnosis later confirmed by identifying pathogenic variants in the *TYR* gene. This case highlights the value of OCT and genetic testing in diagnosing OA, allowing clinicians to provide targeted support for improved visual function and quality of life.

## Introduction

Amblyopia, commonly referred to as “lazy eye,” is a leading cause of preventable visual impairment in children and affects approximately 2% of the population in the United States [1]. Most cases resolve successfully with timely interventions, including refractive correction, patching, and pharmacological therapies [1]. The Pediatric Eye Disease Investigator Group (PEDIG) Amblyopia Treatment Studies (ATS) have shown that timely interventions, such as spectacle correction and occlusion therapy, can significantly improve visual acuity in children [2,3]. Prolonged spectacle wear alone resolves amblyopia in approximately one-third of cases [2]. However, when visual improvement remains limited despite adherence to standard treatment protocols, underlying structural abnormalities should be explored.

Advances in imaging technologies, such as optical coherence tomography (OCT), have revolutionized the evaluation of pediatric patients with refractory amblyopia by enabling detailed visualization of retinal and macular anatomy [4]. Similarly, the increasing accessibility of genetic testing provides clinicians with powerful tools to identify rare hereditary conditions that may impact visual outcomes. This case report highlights the critical role of these diagnostic modalities in uncovering the etiology of atypical amblyopia and emphasizes the importance of a multidisciplinary approach in the evaluation and management of complex pediatric cases.

## Case presentation

A three-year-old blonde male was referred to his pediatrician for a comprehensive eye examination following a failed office vision screening. The patient had no relevant medical history, did not take any medications, and was born full-term via cesarean section. There was no family history of early-onset eye diseases. Upon presentation, the patient's visual acuity was measured at 20/80 in both eyes. There is no nystagmus. Cycloplegic refraction revealed high astigmatism, with a refractive error of -2.25 +3.00 × 98° in the right eye (OD) and -0.50 +3.00 × 90° in the left eye (OS), which the patient accepted without difficulty. The remainder of the exam, including tonometry, anterior segment evaluation, and fundus examination, was unremarkable. The patient was discharged with spectacles and was instructed to wear this full time.

**Figure 1:**
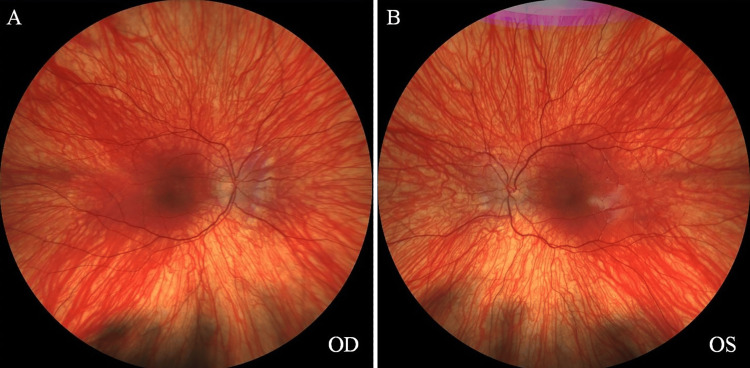
Fundus images of the right eye (OD) and left eye (OS), showing light pigmentation.

Over the next six years, the patient was monitored every six to eight months. The compliance with glasses was intermittent but improved significantly as the child became older. During this time his vision improved slowly and steadily without occlusion therapy or atropine penalization. At the age of nine years, his best spectacle-corrected visual acuity was 20/40 in both eyes, and an inferior oblique muscle overaction in the right eye and exophoria were noted. This was confirmed in two subsequent visits, which also showed stable refraction. The fundus exam is shown in Figures 1A-1B.

**Figure 2:**
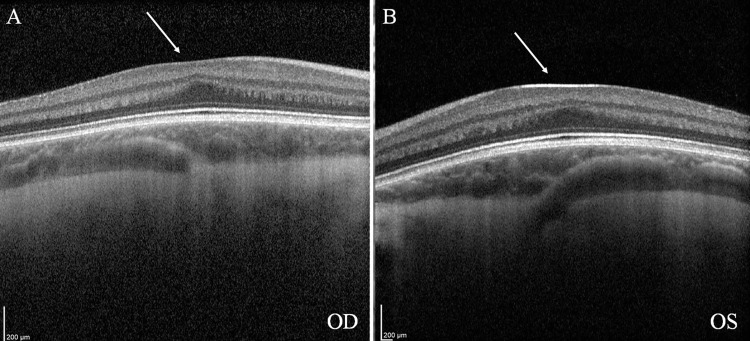
OCT imaging reveals the absence of foveal depression in the right eye (OD) and left (OS) eye (arrows). OCT: Optical coherence tomography

Given the stagnation of visual improvement at 20/40, it was determined that the amblyopia treatment had reached its endpoint. Further investigation was pursued to rule out potential structural abnormalities in the macula. The patient was referred for a neuro-ophthalmology evaluation and underwent OCT imaging, which showed an absence of foveal depression. Figures 2A-2B show a structural anomaly often associated with specific genetic or developmental conditions affecting the retina. Given these findings, the patient and family were counseled on the importance of further genetic testing to clarify the underlying cause of the visual abnormalities. A Molecular Vision Lab® (Hillsboro, Oregon, USA) Vision Panel was subsequently ordered to investigate potential genetic mutations or inherited retinal conditions that could explain the absence of foveal development.

The absence of a foveal depression on OCT indicated a developmental anomaly, such as foveal hypoplasia, where the fovea fails to develop the central depression essential for sharp vision. This finding suggested conditions impacting retinal development and pigmentation when combined with light fundus pigmentation. Ocular or oculocutaneous albinism was the most likely diagnosis, as both conditions are commonly associated with foveal hypoplasia and lighter fundus pigmentation. Other genetic retinal disorders, such as aniridia, congenital stationary night blindness subtypes, or achromatopsia, were also considered but deemed less likely due to the absence of additional distinguishing clinical features, such as iris abnormalities, color vision defects, or photophobia.

Genetic testing results confirmed two pathogenic variants in the *TYR* gene: NM_000372.5(TYR):c.286dupA (p.Met96AsnfsTer73) and NM_000372.5(TYR):c.1217C>T (p.Pro406Leu). These findings confirmed the diagnosis of ocular albinism (OA), bringing resolution to the family’s diagnostic odyssey. Genetic counseling was provided to explain the implications of OA, including its effects on vision and inheritance patterns. Moving forward, the patient will have regular follow-ups to monitor ocular health and refraction, ensuring that visual needs are met and that challenges related to contrast sensitivity or glare are managed proactively.

## Discussion

The diagnosis of foveal hypoplasia secondary to OA in this patient explains the limited improvement in visual acuity despite early refractive correction. Spectacle correction is typically the first-line treatment for amblyopia due to refractive error, and the ATS conducted by the PEDIG showed that children aged three to seven could gain two or more lines of visual acuity with spectacles alone [2,3]. Additionally, about one-third of children with moderate amblyopia (20/40 to 20/100) achieved full resolution with prolonged spectacle wear [2]. However, in cases like this, where underlying structural abnormalities such as foveal hypoplasia are present, visual improvements may be limited due to anatomical barriers affecting central vision. High hyperopia and astigmatism, common in albinism, may also compound these limitations, as refractive errors often correlate with axial length abnormalities [5,6].

Albinism is a rare condition, affecting approximately 1 in 20,000 individuals [7,8]. Albinism is categorized into oculocutaneous albinism (OCA) and OA [5]. OCA involves hypopigmentation of the skin, hair, and eyes with autosomal recessive inheritance, while OA primarily affects the eyes and is X-linked, more commonly affecting males [5]. Albinism disrupts the tyrosine-to-melanin biochemical pathway due to mutations in genes such as *TYR*, *OA1*, and *OCA2* [9]. The most common ocular findings include nystagmus, foveal hypoplasia, iris hypopigmentation and translucency, and retinal hypopigmentation [5,9]. Notably, while nystagmus is common in albinism, about 11% of genetically confirmed patients lack this feature, underscoring the importance of structural and genetic evaluations in cases of atypical amblyopia [10].

In our patient, the visual improvement with spectacle correction and the lack of nystagmus made albinism an unlikely entity on our initial differential diagnoses. An OCT examination of the fundus is typically not offered when a child reaches an amblyopia treatment endpoint. However, OCT imaging revealed abnormal foveal contours bilaterally in our patient, providing an essential clue that guided further genetic evaluation. This case demonstrates the value of OCT in uncovering structural anomalies that may indicate underlying genetic conditions, reinforcing its role in the diagnostic workup of atypical amblyopia. Foveal hypoplasia likely results from reduced tyrosinase activity, which affects cellular development and organization in the retina [11,12]. OCT studies indicate thinning of the outer nuclear layer and increased Henle's fiber layer thickness, likely due to altered foveal cone packing [11,12].

There is currently no cure for albinism [13]. Management of OA requires a comprehensive approach beyond traditional amblyopia treatments. While spectacle correction remains essential, it may not fully address the visual limitations imposed by structural abnormalities. Individualized care should include regular follow-ups to monitor refractive stability, visual development, and strategies to reduce nystagmus intensity. Support groups may provide additional resources to the family when dealing with rare diseases. Additionally, interventions to manage glare and optimize contrast, such as transition lenses or tinted glasses, can be beneficial [13]. Genetic counseling is crucial to educating families about inheritance patterns and prognosis, helping them make informed decisions, and setting realistic expectations for visual outcomes [14].

## Conclusions

This case highlights the importance of OCT and genetic testing in identifying organic causes of atypical amblyopia, such as OA. The absence of foveal depression on OCT and the subsequent confirmation of pathogenic *TYR* mutations provided diagnostic clarity and guided a more tailored approach to management. Recognizing OA enables clinicians to address the condition's physical and psychosocial aspects, ensuring comprehensive care that improves the patient’s quality of life and functional vision. These findings underscore the need for integrating advanced diagnostic tools into clinical practice to enhance outcomes in complex cases of pediatric visual impairment.
